# Association between oral microbial diversity (only bacteria) and diabetes in U.S. adults: analysis of NHANES 2009–2012 data

**DOI:** 10.1186/s12903-025-06204-x

**Published:** 2025-05-28

**Authors:** Shu Yang, Shuo Zhang, Qian Cao, Guowei Zhu, Jikai Liu, Guoqing Li, Minmin Zhu

**Affiliations:** 1 Wuxi School of Medicine, Jiangnan University, Wuxi, China; 2 Tianjin Key Laboratory of Ionic-Molecular Function of Cardiovascular Disease, Department of Cardiology, Tianjin Institute of Cardiology, the Second Hospital of Tianjin Medical University, Tianjin, China; 3 Department of Anesthesiology, Wuxi No.2 People’s Hospital (Jiangnan University Medical Center), Wuxi, China

**Keywords:** Oral microbiome, Diabetes, Microbial diversity, NHANES, A cross sectional study

## Abstract

Studies on the relationship between oral microbial diversity and diabetes were limited. This study analyzed the oral microbial composition and diversity using NHANES data to explore its potential role in diabetes pathogenesis; Methods: A cross-sectional design was employed, utilizing NHANES data (2009–2012), including oral microbiota samples and diabetes-related indicators. Oral samples were collected via mouthwash and analyzed using 16 S rRNA gene sequencing. The Shannon-Wiener Index represented microbial diversity (Only bacteria). Multivariate logistic regression, restricted cubic splines, and subgroup analyses were employed to evaluate associations. Results: A significant association was found between oral microbial diversity and diabetes. According to the completely adjusted model, a one-unit increment in the Shannon-Wiener Index was associated with a 12.1% increase in the likelihood of developing diabetes (OR = 1.121, 95%CI: 1.120 ~ 1.122). Subgroup analyses showed divergent findings. In subgroups with lower body weight and BMI, increased microbial diversity correlated with a decreased likelihood of developing diabetes (OR = 0.68 (0.68–0.68)); Conclusions: Oral microbial diversity exhibits a complex relationship with diabetes risk. The increase and subsequent decrease of oral microbiota diversity in relation to diabetes risk. This suggests that certain specific microbes or interactions between microbes may influence the development of diabetes. However, due to the many limitations of this study, it cannot prove the causal relationship between oral microbial richness and diabetes. Further longitudinal and mechanistic studies are essential to elucidate the causal links and dynamic alterations between the oral microbiome and the progression of diabetes.

## Introduction

Diabetes mellitus, a chronic metabolic disorder characterized by persistent hyperglycemia, represents a major global health concern, affecting millions of individuals worldwide [1]. The prevalence of diabetes is increasing, with type 2 diabetes (T2D) accounting for approximately 90% of all cases [2]. T2D is associated with numerous complications, including cardiovascular disease, kidney failure, and vision loss, which collectively place substantial strain on healthcare systems and the global economy [3].

The oral cavity provides a multifaceted and dynamic environment that hosts various microorganisms, collectively known as the oral microbiome [4]. The health of the mouth is significantly influenced by the composition and regulation of this microbiome, which plays a crucial role in developing oral disorders such as dental caries and periodontal disease [5]. Recent research has also suggested potential links between the oral microbiome and systemic health, particularly in relation to cardiovascular and metabolic diseases [6].

Recent findings indicate a bidirectional relationship between diabetes and oral health. Individuals with diabetes are at an increased risk of developing dental conditions such as periodontitis, due to impaired immune function and changes in the oral environment [7]. Conversely, oral infections and inflammation have been shown to exacerbate insulin resistance, leading to poor glycemic control in diabetic individuals [8]. This interaction underscores the importance of elucidating the underlying mechanisms and potential intermediaries, including the oral microbiome.

Numerous studies have reported changes in the diversity of the oral microbiome in diabetic patients. A particular study [9] found that individuals with type 2 diabetes (T2D) exhibit a distinct oral microbiome profile compared to healthy controls, characterized by an increased presence of specific bacterial species. Similarly, another study [10] identified a link between certain oral microbial species and the risk of developing type 2 diabetes. These findings suggest that alterations in the oral microbiome may serve as potential biomarkers for diabetes, offering valuable insights into the disease’s development. However, relevant meta-analyses demonstrate that current research primarily focuses on the association between specific microorganisms and diabetes, with limited exploration of the link between microbial diversity and diabetes [11, 12].

Nonetheless, the relationship between oral microbial variation and diabetes is complex, and only a limited number of studies have explored this association [9, 13]. We propose that microbial diversity may lead to interactions among microbes, which in turn indirectly affect the impact of microbial communities on diabetes. To address this gap, our study plans to utilize data from the National Health and Nutrition Examination Survey (NHANES) to conduct a cross-sectional analysis examining the relationship between oral microbial diversity and diabetes, while accounting for potential confounding factors. This approach will enhance our understanding of the underlying mechanisms governing this relationship.

## Method

### Data source

The National Center for Health Statistics (NCHS) and the Centers for Disease Control and Prevention (CDC) in the United States conduct NHANES, a continuous national survey that is publicly accessible. A stratified, multi-stage probability sampling approach is used to gather data from civilians who are not institutionalized [14]. The survey data is grouped into two-year periods (e.g., 2009–2010, 2011–2012). NCHS provides survey weights that adjust for selection probabilities and non-response rates, facilitating the production of estimates that represent the national population. The NCHS Institutional Review Board has approved NHANES, and participants have given their informed consent. Johns Hopkins University’s Institutional Review Board has exempted the analysis from review. The survey uses Computer-Assisted Personal Interviewing (CAPI) for home interviews and invites participants to provide additional information at Mobile Examination Centers (MECs), where they undergo medical examinations, participate in further questioning, and provide biological samples. MECs provide a standardized health examination environment, ensuring that measurements and examinations conducted at different locations are consistent. Each MEC is equipped with the most advanced medical devices, such as the Hologic DXA whole-body scanner, which is used to assess obesity and osteoporosis. MECs are also fitted with secure communication equipment that allows for the real-time transmission of data to the NCHS (National Center for Health Statistics). During the examination process, each procedure is explained to the participants by examiners (such as technicians, doctors, phlebotomists, etc.), and participants have the opportunity to ask questions or decline to participate in any procedure. The design and operation of MECs ensure that NHANES can efficiently and standardly collect high-quality health data across the nation, providing important support for public health research and policy-making. Detailed information about the NHANES methodology, including the questionnaires and data collection protocols, can be found on the official NHANES website: https://www.cdc.gov/nchs/nhanes/index.htm.

### Participants

**Fig. 1:**
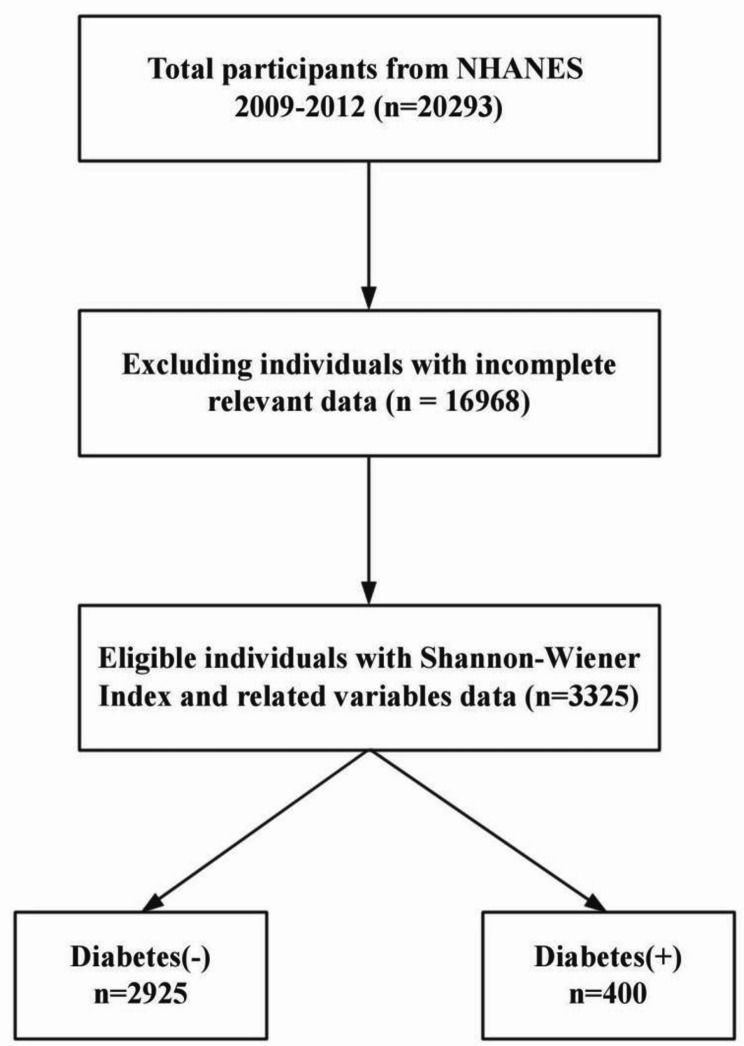
Flowchart of study patients

This study utilized the combined data from the NHANES surveys of 2009–2010 and 2011–2012, including participants who took part in the Mobile Examination Center (MEC) tests and had complete data on the oral microbiome, diabetes, and key covariates of interest (as depicted Fig. 1).

### Oral microbiome measures

Detailed descriptions of the laboratory methods and bioinformatics procedures for NHANES oral microbiome data can be found in the database files on the NHANES website.(https://wwwn.cdc.gov/nchs/data/nhanes/omp/OralMicrobiomeDataDocumentation-508.pdf). The Knight Lab at UC San Diego, California, handled and sequenced the oral rinse specimens. DNA extracted from these samples was used to sequence the V4 region of the 16 S rRNA gene, with data processing conducted using QIIME [15, 16]. DADA2 was employed to generate Amplicon Sequence Variants (Amplicon Sequence Variants (ASVs) are a high-resolution technique for microbiome analysis, used to identify and distinguish different sequence variants within microbial communities. Compared to the traditional Operational Taxonomic Unit (OTU)-based approach, ASVs can detect sequence variations at the single nucleotide resolution, thereby providing higher precision and reliability. The number of ASVs identified can vary depending on the sample type, sequencing depth, and analytical methods used) for 10,442 samples, resulting in a total of 41,378 ASVs [17]. Taxa lacking phylum-level identification (2,750 operational taxonomic units) were excluded from the analysis. Table 1 shows the most common bacterial species in the samples.

**Table 1:** The most common bacterial species in the samples

Bacterial genera	Proportion (Relative Abundance)
Streptococcus	Approximately 20%
Veillonella	Approximately 10%
Actinomyces	Approximately 5%
Prevotella	Approximately 5%
Haemophilus	Approximately 3%
Neisseria	Approximately 3%
Fusobacterium	Approximately 2%
Eubacterium	Approximately 2%
Rothia	Approximately 2%
Others	Approximately 48%

In this study, we used the Shannon-Wiener index to assess species diversity. This index measures diversity within a community by considering both species richness (i.e., the number of species) and evenness (i.e., the relative uniformity of species distribution), calculating the result using the logarithm of species richness and relative abundance [18]. The Shannon-Wiener index is widely applied in ecological research to evaluate species diversity across various ecosystems [19]. The Shannon-Wiener index has become a commonly used diversity metric in ecological research due to its advantages of considering both species richness and evenness, having good mathematical properties, being applicable to various ecosystems, being easy to calculate and interpret, being compatible with other ecological indicators, and having a long history of extensive application. These features enable it to effectively reflect the diversity of ecosystems and provide strong support for ecological research. By comparing the Shannon-Wiener index values across different regions or samples, we can gauge the level of species diversity. Higher values typically indicate greater species diversity. For this study, we utilized a subsampled dataset of 10,000 reads for per subsample and calculated the average from 10 subsamples (The Shannon-Wiener index is calculated based on the relative abundance of different microbial taxa in each sample to assess the diversity of the sample. To ensure a fair comparison among different samples, all sequence data are rarefied to the same depth (e.g., 10,000 sequences per sample) to eliminate the impact of sequencing depth differences on diversity assessment. For different participant groups (e.g., groups with different health statuses or dietary habits), statistical methods such as ANOVA are used to compare the Shannon-Wiener index values to evaluate the differences in diversity among groups).

### Covariates

The covariates included in this study were participants’ age, gender, height, weight, body mass index (BMI), race, education level, hypertension status, smoking status, blood glucose concentration, and blood insulin concentration. Data on age, gender, race, and education level were retrieved from the Demographic Data module. Height, weight, and BMI were collected from the Examination Data module. Blood glucose and insulin concentrations were obtained from the Laboratory Data module. Information on hypertension, diabetes, and smoking status was sourced from the Questionnaire module.

### Statistical analysis

We compiled data from the NHANES cycles of 2009–2010 and 2011–2012. All statistical analyses were performed using appropriate sampling weights. The statistical software employed in this study included SPSS version 26.0 and R version 4.3.3.

In this analysis, normality tests were performed for continuous variables. For data that deviated from a normal distribution, the Wilcoxon rank-sum test was applied, with results presented as the median and interquartile range (IQR). The analysis of categorical variables was conducted with either the chi-square test or Fisher’s exact test, and findings are shown as absolute counts and percentages.

We employed multivariable logistic regression to study the link between diabetes and the diversity of oral microbiota. To explore potential nonlinear relationships, restricted cubic spline (RCS) curves were utilized. Subgroup analyses were carried out to test the robustness of the findings. A *P*-value under 0.05 was regarded as statistically significant.

## Results

### Baseline characteristics

Between 2009 and 2012, a total of 20,293 participants were included in this survey (10,537 in 2009–2010 and 9,756 in 2011–2012), of whom 2,925 had no history of diabetes and 400 had diabetes (Fig. 1). The primary variable of interest, the Shannon-Wiener index, demonstrated a significant difference between the two groups (*P* < 0.001). Additionally, other variables also showed significant differences between the groups (demonstrated in Table 2).

**Table 2:** Baseline information of participants

Variables	Total	Diabetes(-)	Diabetes(+)	*P*-value
	*n* = 3325	*n* = 2925	*n* = 400	
Shannon-Weiner index, M (Q_1_, Q_3_)	4.76 ± 0.64	4.77 ± 0.64	4.69 ± 0.62	< 0.001
Age, M (Q_1_, Q_3_)	43.73 ± 14.22	42.21 ± 13.97	54.83 ± 10.73	< 0.001
Height(cm), M (Q_1_, Q_3_)	168.01 ± 9.92	168.16 ± 9.82	166.86 ± 10.59	< 0.001
Weight(kg), M (Q_1_, Q_3_)	82.46 ± 21.92	81.02 ± 20.94	92.96 ± 25.73	< 0.001
BMI, M (Q_1_, Q_3_)	29.13 ± 6.99	28.56 ± 6.61	33.28 ± 8.17	< 0.001
Insulin(uU/ml), M (Q_1_, Q_3_)	14.80 ± 17.77	14.04 ± 12.37	20.40 ± 38.37	< 0.001
Blood glucose(mmol/L), M (Q_1_, Q_3_)	5.88 ± 1.78	5.56 ± 1.10	8.26 ± 3.35	< 0.001
**Race**,** n(%)**				< 0.001
Mexican American	606 (18.23)	522 (17.85)	84 (21.00)	
Other Hispanic	343 (10.32)	299 (10.22)	44 (11.00)	
Non-Hispanic White	1285 (38.65)	1174 (40.14)	111 (27.75)	
Non-Hispanic Black	713 (21.44)	594 (20.31)	119 (29.75)	
Other Race	378 (11.37)	336 (11.49)	42 (10.50)	
**Gender**,** n(%)**				< 0.001
Female	1706 (51.31)	1509 (51.59)	197 (49.25)	
Male	1619 (48.69)	1416 (48.41)	203 (50.75)	
**Educational level**,** n(%)**				< 0.001
below college	1540 (46.32)	1327 (45.37)	213 (53.25)	
college and above	1785 (53.68)	1598 (54.63)	187 (46.75)	
**Smoking**,** n(%)**				< 0.001
No	1914 (57.56)	1703 (58.22)	211 (52.75)	
Yes	1411 (42.44)	1222 (41.78)	189 (47.25)	
**Hypertension**,** n(%)**				< 0.001
No	2351 (70.71)	2217 (75.79)	134 (33.50)	
Yes	974 (29.29)	708 (24.21)	266 (66.50)	

BMI: Body Mass Index, BMI = weight (kg) / height² (m²)

M: Median, Q_1_: 1st Quartile, Q_3_: 3st Quartile

### Multivariable logistic regression analysis

To investigate the association between oral microbiota diversity and diabetes, we applied three regression models. Model 1 did not include any adjustments, Model 2 accounted for age, gender, height, weight, BMI, and race, while Model 3 included adjustments for all covariates (Table 3). The Shannon-Wiener index was categorized into quartiles to assess differences among groups. In Model 1, which is unadjusted, a one-unit rise in the Shannon-Wiener index corresponded to a 0.079 reduction in diabetes risk (OR = 0.921 [0.920–0.922]). Using Group Q1 as the reference, Group Q2 had the highest risk (OR = 1.127 [1.126–1.128]). In Model 2, which is partially adjusted, a one-unit rise in the Shannon-Wiener index corresponded to a 0.046 increase in diabetes risk (OR = 1.046 [1.046–1.047]). Using Group Q1 as the reference, Group Q2 had the highest risk (OR = 1.595 [1.593–1.597]). According to the fully adjusted Model 3, each additional unit in the Shannon-Wiener index was linked to a 0.121 rise in diabetes risk (OR = 1.121 [1.120–1.122]). Using Group Q1 as the reference, Group Q2 had the highest risk (OR = 1.725 [1.722–1.727]).

**Table 3:** The relationship between Shannon-Weiner index and diabetes (Logistic regression analysis)

Variables	Model 1		Model 2		Model 3	
SWI	HR(95% CI)	*P-value*	HR(95% CI)	*P-value*	HR(95% CI)	*P-value*
Q1 (0.50–4.40)	1 (Reference)		1 (Reference)		1 (Reference)	
Q2 (4.41–4.79)	1.13(1.13 ~ 1.13)	< 0.001	1.60(1.59 ~ 1.60)	< 0.001	1.73(1.72 ~ 1.73)	< 0.001
Q3 (4.79–5.18)	1.07(1.070 ~ 1.07)	< 0.001	1.35(1.35 ~ 1.35)	< 0.001	1.30(1.30 ~ 1.31)	< 0.001
Q4 (5.18–6.60)	0.87(0.87 ~ 0.87)	< 0.001	1.08(1.08 ~ 1.08)	< 0.001	1.18 (1.18 ~ 1.18)	< 0.001

Model 1: No adjusted

Model 2: Adjusted for Age, Gender, Height, Weight, BMI, and Race

Model 3: Adjusted for all covariates

### Restricted cubic spline

**Fig. 2:**
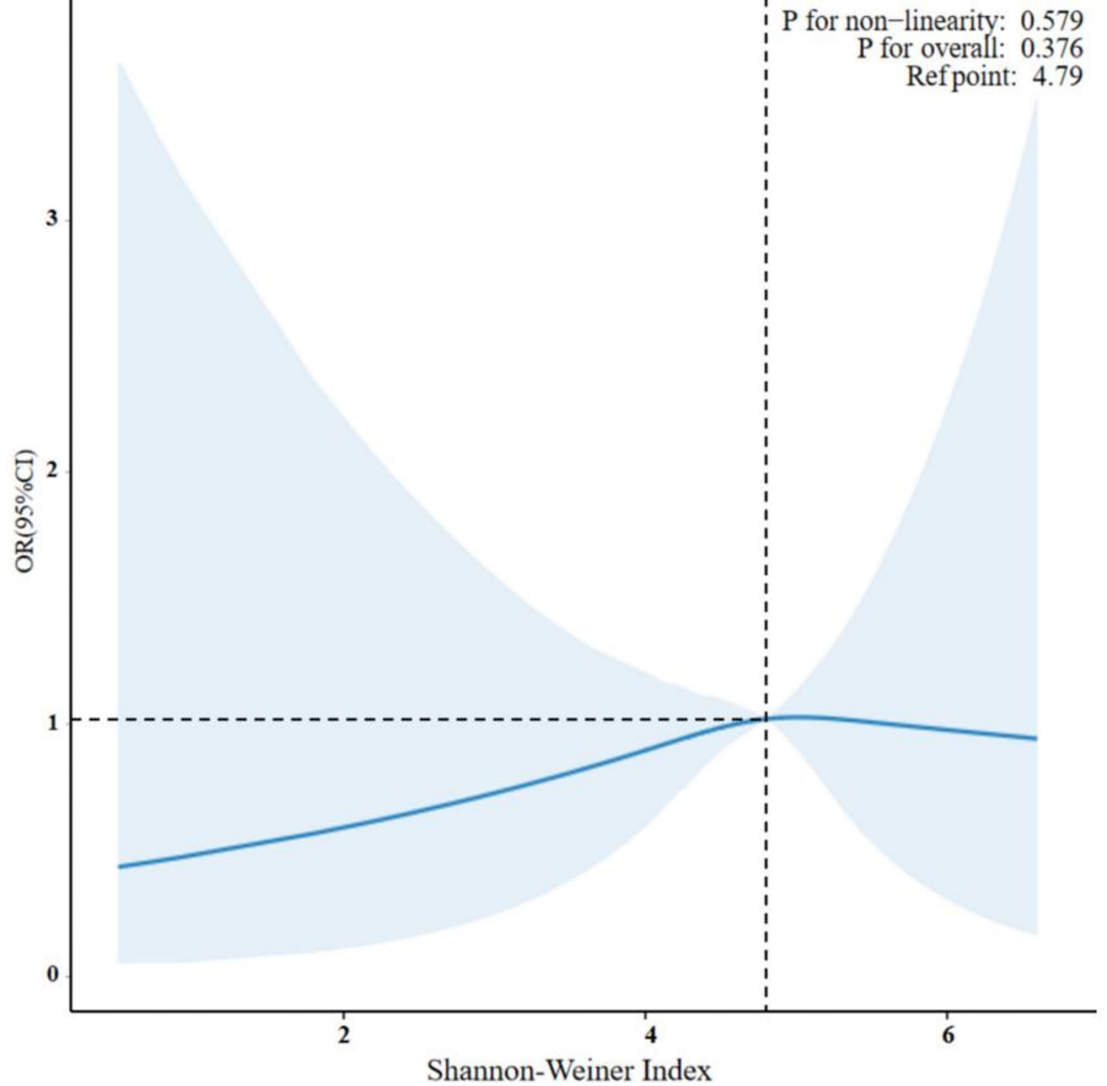
Fitted curve depicting the association between Shannon-Weiner index and diabetes

Restricted cubic spline (RCS) curves were used to investigate the relationship between oral microbiota diversity and diabetes. Figure 2 indicated that as the Shannon-Wiener index increased, the odds ratio (OR) initially increased before decreasing. However, the *p*-value for non-linearity was 0.579, suggesting a linear relationship between the Shannon-Wiener index and the risk of diabetes.

### Subgroup analysis

We conducted a subgroup analysis to assess the robustness of the association between oral microbial species diversity and diabetes (shown in Table 4). The results show that in most subgroups, there is a positive correlation between diabetes risk and oral microbiota diversity. However, in certain specific subgroups (weight < 79.1, BMI < 28), the results differ significantly from those in other subgroups. In these two subgroups, for every one-unit increase in oral microbiota diversity, the risk of diabetes decreases by 32% (OR = 0.68 (0.68–0.68)). This suggests that the interaction between oral microbiota and diabetes may be influenced by complex and multifactorial mechanisms. And this raises the question of whether there are specific environmental factors in these particular subgroups that influence the relationship between microbiota and diabetes.

**Table 4:** Subgroup analysis of the relationship between Shannon-Weiner index and diabetes

Subgroup	Diabetes(+) OR(95%CI)	*P*-Value
**Gender**		
Female	1.07(1.06,1.07)	< 0.001
Male	1.21(1.21,1.22)	< 0.001
**Weight**		
< 79.1	0.68(0.68,0.68)	< 0.001
≥ 79.1	1.32(1.31,1.32)	< 0.001
**Height**		
< 167.7	1.10(1.09,1.10)	< 0.001
≥ 167.7	1.15(1.14,1.15)	< 0.001
**BMI**		
< 28	0.68(0.68,0.68)	< 0.001
≥ 28	1.32(1.32,1.32)	< 0.001
**Educational level**		
college and above	1.10(1.09,1.10)	< 0.001
below college	1.11(1.11,1.11)	< 0.001
**Smoking**		
Yes	1.03(1.03,1.03)	< 0.001
No	1.33(1.33,1.33)	< 0.001
**Insulin**		
< 11.07	0.95(0.95,0.95)	< 0.001
≥ 11.07	1.19(1.19,1.19)	< 0.001
**Blood glucose**		
< 5.5	1.09(1.08,1.09)	< 0.001
≥ 5.5	1.01(1.01,1.01)	< 0.001
**Hypertension**		
Yes	1.24(1.24,1.24)	< 0.001
No	0.98(0.98,0.98)	< 0.001
**Age**		
< 43	1.61(1.61,1.61)	< 0.001
≥ 43	1.08(1.08,1.08)	< 0.001

## Discussion

The nationally representative data presented in this study suggest an association between oral microbiome diversity and diabetes risk. In the adjusted analysis, as species diversity increases, the risk of developing diabetes first rises and then declines, with the highest risk observed in the Q2 group. However, in certain subgroups (Weight < 79.1, BMI < 28), an increase in species diversity is significantly negatively correlated with the risk of diabetes, indicating that different mechanisms may be involved in these subgroups.

We speculate that oral microbiota may interact with each other and be associated with diabetes through the following mechanisms. The oral microbiome can influence energy homeostasis by modulating host metabolism. Short-chain fatty acids (SCFAs), produced by oral microorganisms, have been shown to stimulate the secretion of glucagon-like peptide-1 (GLP-1) and peptide YY (PYY), thereby enhancing insulin production and reducing hunger, respectively [20–22]. Persistent low-grade inflammation is a common feature of both diabetes and periodontal disease. Oral microorganisms contribute to systemic inflammation through the secretion of pro-inflammatory cytokines and lipopolysaccharides (LPS), leading to reduced insulin sensitivity and exacerbating diabetes progression [23]. Additionally, the oral microbiome can modulate the host’s immune response, influencing the balance between inflammatory and anti-inflammatory reactions. For example, certain microorganisms may activate regulatory T cells (Tregs) to mitigate inflammation, thereby improving insulin sensitivity [24]. Moreover, the oral microbiome can affect the hypothalamic-pituitary-adrenal (HPA) axis, which regulates the stress response. Chronic stress elevates cortisol levels, potentially impacting insulin sensitivity and promoting diabetes onset. By modulating the HPA axis, oral microbes may influence stress responses and glucose metabolism [25, 26]. Some studies have found that certain specific taxonomic units within the oral microbiota are associated with the risk of type 2 diabetes. For example, the relative abundance of the phylum Actinobacteria is significantly reduced in individuals with diabetes, while certain specific genera, such as Actinomyces and Atopobium, are associated with a decreased risk of diabetes [9]. Additionally, other microbes may indirectly influence diabetes by affecting certain diabetes-related microbes. However, the above are the possible mechanisms by which microbial communities influence diabetes, and the exact mechanisms need to be further explored by us.

In the subgroups with lower body weight and BMI, we found that the relationship between oral microbiota richness and diabetes was quite different from the results in other subgroups. We propose several potential explanations for these findings. Individuals with lower body weight (< 79.1 kg) and BMI (< 28) exhibit a significantly different metabolic profile compared to those who are overweight or obese. Specifically, these individuals tend to have less adipose tissue, and their adipokine secretion patterns differ from those observed in obese individuals. Adipokines play a critical role in regulating insulin sensitivity and glucose metabolism, and oral microbiota may interact with these distinct physiological processes, potentially influencing diabetes risk in a manner that differs from the general population [27, 28]. In specific subgroups characterized by lower body weight and body mass index (BMI), the oral microbiota may display distinct structural and compositional features. Particular bacterial species or combinations of microbial communities within these individuals could be significantly enriched. These microbes may produce bioactive metabolites with metabolic regulatory functions, such as short-chain fatty acids and polyamines, or activate immune-modulatory pathways, thereby enhancing insulin sensitivity, optimizing glucose metabolism regulation, and potentially reducing the risk of diabetes.

These findings highlight the intricate interplay between oral microbiome diversity and the onset of diabetes. However, our study cannot prove a causal relationship between microbial diversity and diabetes. Longitudinal studies are essential to establish causality and investigate the dynamic changes in the oral microbiome over time in relation to diabetes development. Furthermore, mechanistic studies are critical for elucidating the specific pathways by which oral microbiota influence glucose metabolism and insulin sensitivity. This may involve both in vitro and in vivo experiments to examine how specific oral microbes or metabolites affect pancreatic β-cell function, insulin signaling, and systemic inflammation. A deeper understanding of how oral microbiome diversity impacts diabetes risk could have significant clinical implications. If these findings are confirmed, the oral microbiome could serve as a potential biomarker for early detection of diabetes risk. Interventions aimed at modulating the oral microbiome, such as probiotics or prebiotics, might emerge as strategies for diabetes prevention or management, particularly for high-risk subgroups identified in this study.

In contrast to prior studies that concentrated on specific microbial communities, our study highlights the diversity of the oral microbiome to investigate its potential link with diabetes. The analysis leverages the NHANES dataset, which not only provides a substantial sample size but also extensively captures the heterogeneity of the US population, encompassing factors such as age, gender, race, and geographical location, thereby ensuring robust sample representation. Moreover, by incorporating and adjusting for pertinent variables, this study successfully mitigates the impact of certain confounding factors on the outcomes, further strengthening the reliability and scientific validity of the conclusions.

Ultimately, this research provides critical insights into the correlation between oral microbiome diversity and diabetes risk, underscoring the need for further studies to elucidate the underlying mechanisms and explore potential therapeutic applications. Such investigations may enhance our understanding of disease progression in the context of oral microbiome diversity. Continued efforts are essential for exploring potential causative links and identifying specific bacterial taxa or processes driving these associations.

## Conclusion

The study reveals a multifaceted relationship between oral microbial diversity and diabetes risk. The increase and subsequent decrease of oral microbiota diversity in relation to diabetes risk, but this correlation does not uniformly apply to all subgroups. In certain subgroups, increased diversity correlates with a lower risk, indicating that complex mechanisms govern the relationship between oral microbes and diabetes. This suggests that certain specific microbes or interactions between microbes may influence the development of diabetes. Further longitudinal and mechanistic studies are needed to establish cause and effect and to investigate the dynamic changes in the oral microbiome over time in relation to diabetes progression.

### Limitations

This study is a cross-sectional investigation, which, despite its capacity for swiftly delineating the distribution of variables and their associations, harbors several limitations. It struggles to establish a causal link between exposure and outcome. Since NHANES does not systematically distinguish between type 1, type 2, or gestational diabetes during its data collection process, we were unable to incorporate analyses specific to different diabetes types. Moreover, although this study adjusted for certain confounding factors, it could not incorporate all relevant variables due to various constraints, such as participants’ dietary habits and oral health status. Use of 16 S rRNA sequencing limits functional insights Additionally, the data from NHANES are derived solely from the U.S. population, precluding the generalization of the study’s findings to populations in other countries.

## Data Availability

The data used in this study are publicly available from the National Health and Nutrition Examination Survey (NHANES) website (https://www.cdc.gov/nchs/nhanes.htm). The sequencing data of oral microorganisms are publicly available under the accession number PRJNA896165 and can be accessed via the following link: https://www.ncbi.nlm.nih.gov/sra.

## Abbreviations

95%CI: 95% Confidence Interval
BMI: Body Mass Index
M: Median, Q_1_:1st Quartile, Q_3_:3st Quartile
OR: Odds Ratio
SWI: Shannon-Weiner index
